# Enhancing the solubility of daidzein in soybean oil via ultrasound-assisted magnetic stirring: Impacts on oil storage stability and thermal loss during French fries preparation

**DOI:** 10.1016/j.fochx.2026.103987

**Published:** 2026-05-15

**Authors:** Tan Wang, Siwei Chen, Wenxi Li, Maofeng Dong, Shiquan Shen, Mingfu Wang, Hui Wang, Yueliang Zhao

**Affiliations:** a School of Public Health, Shanghai Jiao Tong University School of Medicine, Shanghai 200025, China; b Institute for Agro-food Standards and Testing Technology, Shanghai Academy of Agricultural Sciences, Shanghai 201106, China; c College of Food Science and Technology, Shanghai Ocean University, Shanghai 201306, China; d Tea Research Institution, Yunnan Academy of Agricultural Sciences, Kunming 650200, Yunnan, China; e Shenzhen Key Laboratory of Food Nutrition and Health, College of Chemistry and Environmental Engineering, Shenzhen University, Shenzhen 518060, China

**Keywords:** Daidzein, Ultrasound-assisted magnetic stirring, Storage stability, French frying model, Thermal loss

## Abstract

Daidzein's extremely low solubility in edible oils limits its application. This study developed a green, solvent-free ultrasound-assisted magnetic stirring strategy to enhance daidzein dissolution in soybean oil. The optimized process increased the dissolved daidzein concentration to a thermodynamic saturation limit of 58.12 mg/kg. While the absolute concentration is modest, the resulting fortified oil exhibited markedly improved storage stability over six months, effectively retarding deterioration in color, acid value, and peroxide value, and better preserving unsaturated fatty acids. Additionally, a simulated French fry model revealed that daidzein retention decreased with increasing temperature and frying duration. This thermal loss was governed by three contributing mechanisms: volatilization, chemical transformation (yielding an unidentified daidzein-derived product), and migration into the food matrix. These findings provide a theoretical foundation for developing daidzein-fortified oils and optimizing thermal processing conditions to retain valuable bioactive components.

## Highlights

• An ultrasound-magnetic stirring method enhanced daidzein solubility in soybean oil.
• Daidzein fortification significantly improved oil oxidative stability during storage.
• Fortified oil better preserved color, acidity, peroxide value, and unsaturated fatty acids.
• Thermal loss of daidzein was caused by volatilization, transformation, and migration.
• A novel daidzein-fatty acid derivative was identified in heated oil.

## 1 Introduction

Soybean oil is valued for its favorable nutritional profile, characterized by a high concentration (nearly 80%) of unsaturated fatty acids, primarily oleic, linoleic, and linolenic acids, alongside bioactive phytochemicals such as plant sterols and carotenoids (Block et al., 2022; Orsavova et al., 2015). This composition underpins several health benefits: unsaturated fatty acids contribute to cardiovascular health by modulating blood lipids; plant sterols competitively inhibit intestinal cholesterol absorption; and carotenoids provide antioxidant activity (Swallah et al., 2023). Driven by growing consumer interest in preventive health, the food industry is increasingly focused on functional foods, with significant opportunities in the fats and oils sector. While current innovations often target fatty acid optimization (Li et al., 2022), a persistent technical challenge is the effective incorporation of poorly oil-soluble bioactive compounds, such as the isoflavone daidzein, into edible oils.

Daidzein, a major phytoestrogenic isoflavone found predominantly in soybeans and soy products, exhibits a broad spectrum of biological activities, including antioxidant, anti-tumor, anti-cardiovascular disease, and anti-inflammatory effects (Ahmad et al., 2024; Lee et al., 2003). Its natural concentration in soybeans is variable (1–220 mg/kg), influenced by agronomic and geographical factors (Erdoğan Orhan et al., 2007; Lee et al., 2003). However, due to the inherently low oil solubility of isoflavones, most daidzein is lost with processing by-products such as okara during conventional oil extraction. Consequently, commercial soybean oil contains only trace amounts, typically ranging from 0.08 to 1.47 mg/kg (Zhao et al., 2015). Enriching soybean oil with its native isoflavone daidzein offers a clean-label strategy that aligns with consumer preference for naturally sourced ingredients. Moreover, such fortified oil provides dual functionality, stabilizing the oil against oxidation while potentially contributing phytoestrogenic bioactivity, without the need for synthetic additives or organic solvents. This approach also supports circular nutrition by recovering and reusing a bioactive component naturally present in soybeans, rather than discarding it with processing by-products. For consumers who regularly use soybean oil as their primary cooking oil, this fortification offers a seamless way to increase isoflavone intake without changing dietary habits. However, the direct addition of daidzein to oil is severely hindered by its extremely low solubility. To address this challenge, strategies like esterification and co-dissolution with ethanol have been explored (Vojvodić et al., 2023; Wang et al., 2023; Zhao et al., 2022). Nevertheless, esterification can diminish antioxidant potency by masking hydroxyl groups (Liu et al., 2021; Zhang et al., 2021), while ethanol use raises concerns about residual solvent in the final product (Vojvodić et al., 2023). Therefore, developing efficient, green, and solvent-free physical methods to enhance daidzein solubility in oil is a compelling alternative.

The practical application of daidzein as an oil additive depends critically on two factors: its ability to improve the oxidative stability of the oil during storage, and its own stability under storage and thermal processing conditions. Previous studies indicate that certain natural polyphenols, including quercetin, catechin, and gallic acid, can significantly enhance the storage and thermal stability of various edible oils (Liu et al., 2021; Wu et al., 2019; Zhang et al., 2021). In contrast, other polyphenols like rutin and luteolin 7-O-glucoside degrade rapidly upon heating, with retention falling below 10% and 20%, respectively, after just 1 h at 110 °C, limiting their utility (Chaaban et al., 2017). Compounds such as quercetin and naringin demonstrate greater thermal resilience. They retain over 25% of their original content after heating in soybean oil at 180 °C for 4 h, and 80% after heating at 110 °C for 1 h, making them promising candidates for thermal processing (Chaaban et al., 2017; Zhang et al., 2021). Despite this context, the specific effects of daidzein on soybean oil stability and its own thermal behavior remain poorly characterized, with no quantitative data on its thermal loss kinetics, migration into the food matrix, or the structural fate of transformation products during repeated frying cycles, representing a significant knowledge gap.

In this study, we developed a daidzein-fortified soybean oil. To overcome the solubility limitation, a solvent-free physical method combining ultrasound and magnetic stirring was employed. We systematically evaluated the impact of daidzein fortification on the storage stability of soybean oil. Furthermore, using a French fry frying model, we investigated the thermal loss of daidzein and elucidated its underlying mechanisms. This work aims to establish a theoretical foundation for the development of daidzein-enriched soybean oil products.

## 2 Materials and methods

### 2.1 Chemicals and reagents

Refined soybean oil was purchased from Shanghai Titan Technology Co., Ltd. (Shanghai, China). Prior to delivery, the supplier removed natural minor components using conventional chromatographic techniques and vacuum-packed the oil. Daidzein (purity ≥95%) was obtained from Sigma-Aldrich (St. Louis, MO, USA). Potatoes for French fry preparation were procured from a local supermarket in Shanghai. A certified reference mixture of 37 fatty acid methyl esters (FAMEs) and nonadecanoic acid methyl ester (C19:0, internal standard) were acquired from Shanghai ANPEL Scientific Instrument Co., Ltd. (Shanghai, China). HPLC-grade solvents, including acetonitrile, *n*-hexane, methanol, and formic acid, were supplied by Merck KGaA (Darmstadt, Germany). All other chemicals and reagents were of analytical grade and sourced from Shanghai ANPEL Scientific Instrument Co., Ltd.

### 2.2 Preparation of daidzein-fortified soybean oil

**Fig. 1:**
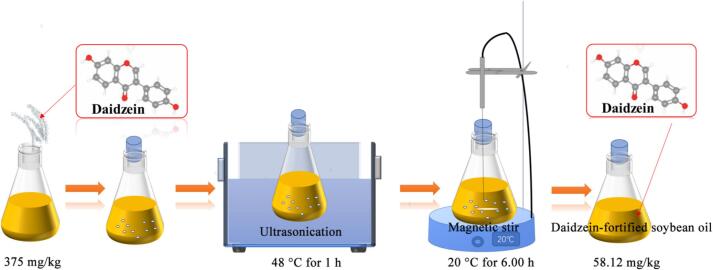
Steps of ultrasound-assisted magnetic stirring assisted dissolution of daidzein in soybean oil.

A combined physical method employing ultrasound-assisted magnetic stirring was used to enhance the solubility of daidzein in soybean oil (Fig. 1). Key processing parameters, including initial daidzein addition, ultrasonication duration and temperature, as well as magnetic stirring duration and temperature, were first screened through single-factor experiments. In these screening experiments, the initial daidzein addition ranged from 250 to 400 mg/kg. Ultrasonication was performed using a fixed power of 300 W and a frequency of 40 kHz, with durations varying from 0.5 to 2 h and temperatures from 20 to 50 °C. Subsequent magnetic stirring was conducted at a constant speed of 800 rpm, for 2 to 8 h at 20 to 50 °C. These parameters were subsequently optimized using a three-factor, three-level Box-Behnken design (BBD) coupled with response surface methodology (RSM) to maximize dissolution. The optimized procedure was as follows: a predetermined mass of daidzein was added to soybean oil. The mixture was first subjected to ultrasonication in a bath to disperse and reduce the particle size of daidzein. It was then immediately transferred to a magnetic stirrer for further agitation to promote dissolution. Finally, the mixture was centrifuged at 4000 rpm for 5 min and filtered through a qualitative filter paper to remove any undissolved particulates, yielding a clear, daidzein-fortified soybean oil with a defined concentration.

### 2.3 Microscopic analysis of oil samples

The efficacy of the solubilization process was visually assessed using an inverted optical microscope (EVOS, Thermo Fisher Scientific, USA). Three sample types were examined: (A) plain soybean oil (control), (B) soybean oil with daidzein added but without any physical treatment (untreated mixture), and (C) daidzein-fortified oil prepared under the optimal ultrasound-magnetic stirring conditions. Images were captured to compare the presence and dispersion of undissolved daidzein particles.

### 2.4 Analysis of color, acid value, and peroxide value

Oil samples were stored in amber glass bottles at room temperature (25 ± 2 °C) for six months. The bottles were intentionally not flushed with nitrogen to better simulate standard consumer storage conditions and allow natural autoxidation to occur. Our sealed amber-bottle design does not fully simulate the repeated opening/closing and oxygen exposure typical of consumer use. A 90-day interval for analyzing color, acid value (AV), and peroxide value (PV) was chosen because lipid autoxidation at room temperature is a relatively slow process. Color parameters (L*,* a, and b) were measured using a Lovibond PFX-1 Series colorimeter (The Tintometer Ltd., UK). AV and PV were determined following the AOCS Official Methods Cd 3d-63 and Cd 18–90, respectively (AOCS, 2017). All analyses were performed in triplicate.

### 2.5 Fatty acid composition analysis by gas chromatography (GC)

Fatty acids were derivatized to their methyl esters (FAMEs) according to a method adapted from Kong et al. (2023). Briefly, 100 mg of oil was mixed with 100 μL of C19:0 internal standard solution (10 mM in hexane). After adding 5 mL of 0.5 M methanolic NaOH, the mixture was vortexed and heated at 100 °C for 5 min following the addition of 3 mL of 14% boron trifluoride-methanol (*w*/w). After cooling, 2 mL of *n*-hexane was added, and the mixture was reheated at 100 °C for 3 min. Subsequently, 8 mL of saturated NaCl solution was added. The mixture was centrifuged (4000 rpm, 5 min), and the upper hexane layer was filtered through a 0.22 μm PTFE membrane for GC analysis.

GC analysis was performed on a Thermo Fisher Trace 1310 system (Waltham, MA, USA) equipped with a flame ionization detector (FID) and an Agilent SP-2560 fused silica capillary column (100 m × 0.25 mm × 0.20 μm). Nitrogen was used as the carrier gas at a constant flow of 1.0 mL/min. The injector and detector temperatures were set at 250 °C and 260 °C, respectively. The oven temperature program was: 70 °C (hold 1 min), increase to 140 °C at 20 °C/min (hold 1 min), then to 180 °C at 4 °C/min (hold 1 min), and finally to 225 °C at 3 °C/min (hold 30 min). FAMEs were identified by comparing retention times with authentic standards and quantified relative to the internal standard (C19:0) peak area. Results are expressed as g per 100 g of oil (g/100 g).

### 2.6 HPLC analysis of daidzein content

The daidzein content in oil was determined by HPLC following a modified extraction procedure (Erdoğan Orhan et al., 2007). A 2.00 g (±0.01 g) of oil was weighed into a 50 mL centrifuge tube. Then, 10 mL of n-hexane and 10 mL of 90% aqueous methanol (*v*/v) were added. The mixture was vortexed vigorously for 3 min to partition daidzein into the methanolic phase. After centrifugation at 1200 rpm for 3 min, the upper hexane layer was discarded. The methanolic extraction was repeated twice. The combined methanolic phases were adjusted to a final volume of 20 mL with 90% methanol, filtered through a 0.22 μm nylon membrane, and analyzed by HPLC. Chromatographic separation was achieved on a YMC-Pack ODS-A C18 column (5 μm, 250 mm × 4.6 mm i.d.) maintained at 30 °C. The mobile phase consisted of (A) 0.1% acetic acid in water and (B) 0.1% acetic acid in acetonitrile, using a gradient elution: 0–12.5 min, 10–30% B; 12.5–17.5 min, 30–40% B; 17.5–18.5 min, 40–100% B; 18.5–21.0 min, 100% B; 21.0–22.5 min, 100–10% B; 22.5–26.0 min, 10% B for column re-equilibration. The flow rate was 1.0 mL/min, and detection was performed at 254 nm. To accurately quantify daidzein in soybean oil while compensating for potential matrix effects and extraction losses, a matrix-matched calibration curve was employed in place of a pure solvent-based standard curve. A daidzein stock solution (1.0 mg/mL) was first prepared in HPLC-grade methanol. For matrix-matched calibration, appropriate volumes of daidzein working solutions were spiked into blank soybean oil (free of daidzein) to achieve theoretical concentrations spanning the expected sample range (0, 10, 20, 50, 100, and 150 mg/kg). These spiked oil samples were then subjected to the same liquid-liquid extraction procedure described in Section 2.6 (using n-hexane and 90% aqueous methanol), and the resulting methanolic extracts were analyzed by HPLC. A calibration curve was constructed by plotting peak area against the theoretical daidzein concentration in oil, and the daidzein concentrations in actual oil samples were subsequently calculated by interpolating their peak areas onto this matrix-matched external standard calibration curve, thereby effectively compensating for variations in extraction efficiency and matrix effects inherent to the oil matrix (Wang et al., 2024).

### 2.7 French fries frying model

A controlled experimental batch-frying model was established to evaluate thermal processing under standardized conditions, adapted from Shah et al. (2025). Briefly, fresh potatoes were cut into uniform strips (approx. 10 × 10 × 50 mm), rinsed, and patted dry. For each frying experiment, oil (either fortified or unfortified control) was placed in an open glass flask equipped with a thermal sleeve for temperature control. Frying was conducted at four distinct temperatures: 140, 160, 180, and 200 °C (± 5 °C). The oil-to-potato ratio was fixed at 10:1 (*w*/w). The total cumulative heating time for each oil batch was 8 h, structured to simulate intermittent use: this period comprised three separate “batch” cycles, with a 1-h idle period at the target temperature between cycles. Within each 2-h active batch cycle, ten consecutive frying sessions were performed, each involving frying potato strips for 3 min followed by a 10-min oil recovery interval. No fresh oil was replenished during the entire process to isolate the effect of repeated heating. Oil and French fry samples were collected at predetermined intervals corresponding to specific cumulative frying times (e.g., after batch 1, 10, 11, 20, 21, and 30 frying sessions). Collected French fries were immediately freeze-dried, ground into a fine powder, and stored at −20 °C until analysis.

### 2.8 Analysis of daidzein transformation products by UPLC-Q-TOF-MS/MS

To investigate thermal transformation, daidzein-fortified oil was heated at 180 °C for 8 h in the absence of food. For analysis, 800 μL of heated oil was mixed with 1600 μL of methanol, vortexed for 2 min, and centrifuged. The supernatant was filtered (0.22 μm organic solvent membrane) prior to UPLC-MS analysis. Separation was performed on an Agilent 1290 UPLC system using a Waters BEH C18 column (1.7 μm, 2.1 × 100 mm). The column temperature was 35 °C*. mobile* phases were (A) 0.1% formic acid in water and (B) acetonitrile. A gradient elution was applied (0–0.5 min, 2% B, 0.5–3 min, 2–70% B; 3–10 min, 70–98% B; 10–12 min, 98% B; 12–14 min, 98–2% B; 14–16 min, 2% B) at a flow rate of 0.3 mL/min. The injection volume was 5 μL. Mass spectrometry was conducted on an Agilent 6550 Q-TOF spectrometer with electrospray ionization in negative mode (ESI-). Key parameters were: capillary voltage, 3.2 kV; fragmentor voltage, 35 V; collision energy, 6 eV; source temperature, 125 °C; desolvation gas (N2) flow, 12 L/min at 350 °C. Data were acquired in centroid mode over an *m*/*z* range of 50–1000.

### 2.9 Analysis of daidzein migration into French fries

Daidzein migrated into French fries was extracted and quantified. Freeze-dried fry powder (0.5 g) was mixed with 5 mL of methanol and sonicated for 30 min. After centrifugation (3000 rpm, 5 min), the supernatant was collected. The extraction was repeated twice. The combined supernatants were evaporated to dryness at 40 °C under nitrogen. The residue was reconstituted in 2 mL of acetonitrile and washed twice with 3 mL of n-hexane (vortexing and centrifugation at 2000 rpm for 3 min) to remove co-extracted lipids. The final acetonitrile phase was evaporated, reconstituted in 2 mL methanol, filtered (0.22 μm), and analyzed by HPLC using an isocratic method (methanol: water = 60:40, *v*/v) at a flow rate of 0.6 mL/min over 20 min, with detection at 254 nm. Daidzein was identified by comparing its retention time with that of standard, and quantification was carried out using the external standard method.

### 2.10 Microstructure analysis of French fry crust by scanning electron microscopy (SEM)

The surface microstructure of French fries fried at different temperatures was examined by SEM (ZEISS Gemini 300, Germany). Small sections of the fry crust were carefully excised, dehydrated, and defatted using standard protocols. The samples were then sputter-coated with gold and observed under the SEM at an accelerating voltage of 10 kV and a magnification of 100 × .

### 2.11 Statistical analysis

All experiments were conducted with three independent replicates. Data are presented as mean ± standard error of the mean. Statistical analysis was performed using IBM SPSS Statistics 25.0. Differences between means were evaluated by Student's *t*-test or one-way analysis of variance (ANOVA) followed by Tukey's post-hoc test, as appropriate. Significance was defined at *p* < 0.05. The RSM design and analysis were performed using Design-Expert software (version 13, Stat-Ease Inc., USA). Graphical presentations were prepared using GraphPad Prism 8.0 (GraphPad Software, San Diego, CA, USA).

## 3 Results and discussion

### 3.1 Effect of ultrasound-assisted magnetic stirring on the solubility of daidzein in soybean oil

The solubility of daidzein in soybean oil was enhanced by employing a combined physical method of ultrasound-assisted magnetic stirring (Fig. 1). This approach leverages complementary mechanisms: ultrasonic cavitation promotes particle size reduction and accelerates mass transfer, thereby facilitating dissolution (Li et al., 2022), while subsequent magnetic stirring ensures homogeneous dispersion of the finely divided particles throughout the oil matrix (Cheng et al., 2023). To establish the optimal processing conditions, a systematic optimization strategy was employed, beginning with one-factor-at-a-time experiments followed by response surface methodology (RSM).

Single-factor experiments investigated the individual effects of key parameters, including ultrasonication time (0.5–2 h), ultrasonication temperature (20–50 °C), magnetic stirring time (2–8 h), magnetic stirring temperature (20–50 °C), and the initial amount of daidzein added. The intrinsic (unassisted) solubility of daidzein in the soybean oil was determined to be 0.51 mg/kg. For the optimization trials, daidzein was added to the oil at a loading of 200 mg/kg prior to treatment. As illustrated in Fig. S1A-E, three parameters, ultrasonication temperature (Fig. S1B), magnetic stirring time (Fig. S1C), and initial daidzein addition amount (Fig. S1E), exerted marked effects on solubility. In contrast, the effect of ultrasonication time plateaued beyond 1 h (Fig. S1A), and magnetic stirring temperature showed negligible influence (Fig. S1D). Each of the three key factors exhibited a nonlinear relationship with solubility, reaching an optimum before declining. Therefore, ultrasonication temperature, magnetic stirring time, and daidzein addition amount were selected for further optimization via response surface methodology (RSM) to elucidate their interactions and identify optimal levels.

In the RSM design, the dissolved daidzein concentration (Y, mg/kg) served as the response variable. The independent variables were defined as initial daidzein addition amount (X_1_), ultrasonication temperature (X_2_), and magnetic stirring time (X_3_). A three-factor, three-level Box-Behnken design was implemented (Table S1a). The relationship between the response and the independent variables was modeled by the following second-order polynomial equation: Y = 58.708 + 0.71 × _1_ + 2.05375 × _2_ + 0.85125 × _3_–1.3825X_1_X_2_–1.355X_2_X_3_–7.9115 × _1_^2^–1.034 × _2_^2^–1.329 × _3_^2^. The magnitude and sign of each coefficient indicated the effect size and direction of the corresponding factor or interaction. The analysis of variance (ANOVA) for the quadratic model (Table S1b) confirmed that the model was highly significant (*p* < 0.0001) with a non-significant lack of fit (*p* = 0.1424), demonstrating good fit to the experimental data. The high coefficient of determination (R^2^ = 0.9831) and the low coefficient of variation (CV = 1.60%) further validated the model's reliability and predictive capability for daidzein solubility under the studied conditions. The ANOVA results further elucidated the relative influence of the model terms. The factors and their interactions showed varying significance levels: X_2_ and X_1_^2^ exerted highly significant effects (*p* < 0.01), while X_1_, X_3_, X_1_X_2_ and X_2_X_3_, X_2_^2^ and X_3_^2^, were significant (*p* < 0.05). Based on the magnitude of the effects, the order of influence on daidzein solubility was X_2_ > X_3_ > X_1_. The interaction effects were visualized in the response surface and contour plots (Fig. S1F—I). Fig. S1F-G showed that at a fixed ultrasonication temperature, daidzein solubility first increased and then decreased with higher daidzein addition, while at a fixed daidzein level, solubility rose steadily with increasing temperature. The steep, elliptical contours confirmed a significant interaction between these two factors. Similarly, Fig. S1H-I indicated that solubility increased with either higher ultrasonication temperature (at constant stirring time) or longer magnetic stirring (at constant temperature), again supported by the curved surface and elliptical contours, reflecting a clear interaction between ultrasonication temperature and magnetic stirring time.

**Fig. 2:**
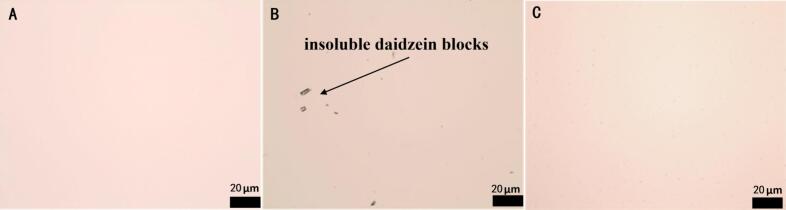
Representative light micrographs of: (A) plain (unfortified) soybean oil (control); (B) Soybean oil with daidzein added (58 mg/kg) but without any physical solubilization treatment; (C) Daidzein-fortified soybean oil (58 mg/kg) prepared under the optimized ultrasound-assisted magnetic stirring conditions.

The model predicted the optimal dissolution conditions to be: daidzein addition of 373.63 mg/kg, ultrasonication at 47.09 °C for 1 h, and magnetic stirring for 5.96 h, yielding a predicted dissolved daidzein concentration of 58.75 mg/kg. For operational feasibility and instrument set-point precision during actual laboratory preparation, these conditions were slightly adjusted to 375.00 mg/kg, 48.00 °C for 1 h, and 6 h at 20 °C, respectively, with a corresponding predicted yield of 58.68 mg/kg. Experimental validation under these adjusted conditions achieved a dissolved daidzein concentration of 58.12 mg/kg, demonstrating excellent agreement with the model prediction and representing a solubility increase of over 110-fold compared to the untreated oil. Crucially, the undissolved daidzein (316.88 mg/kg) is not lost; it is separated via simple centrifugation/filtration (described in Section 2.2). This residue can be recovered and is expected to be recyclable, pending further empirical validation, thereby minimizing material waste. Visual evidence supported the quantitative results. As shown in Fig. 2, the daidzein-fortified oil prepared under optimal conditions (Fig. 2C) was clear and transparent, indistinguishable in appearance from the plain soybean oil control (Fig. 2A). In stark contrast, oil containing an equivalent amount of daidzein but prepared without physical assistance (Fig. 2B) exhibited visible, undissolved particulate matter. This confirms that the ultrasound-assisted magnetic stirring method not only dramatically enhances dissolution but also ensures a homogeneous distribution of daidzein within the oil.

Collectively, the synergistic application of ultrasonication and magnetic stirring proved to be a highly effective, solvent-free physical strategy for incorporating daidzein into soybean oil. The success of the RSM optimization underscores the method's controllability and efficiency. By achieving significant solubility enhancement without chemical modification or organic solvents, this approach addresses key technical and safety concerns associated with fortifying oils with lipophobic bioactive compounds. Given daidzein's recognized bioactivity (Ahmad et al., 2024; Lee et al., 2003), this method establishes a viable foundation for developing daidzein-enriched soybean oil products.

### 3.2 Effect of daidzein on the storage stability of soybean oil

#### 3.2.1 Color stability

**Fig. 3:**
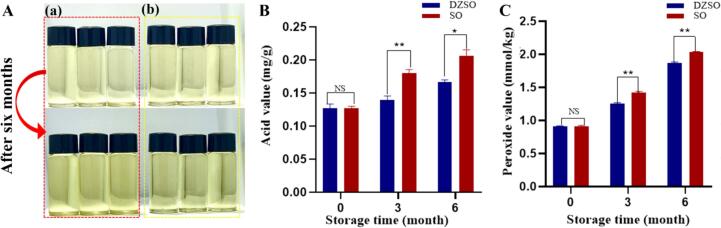
Changes in physicochemical stability indices of plain and daidzein-fortified soybean oil during six months of storage. (A) Visual appearance of oil samples immediately after preparation (top row) and after six months of storage (bottom row): plain soybean oil (left) and daidzein-fortified soybean oil (right). Images are representative of three replicates. (B) Acid value and (C) Peroxide value of oil samples over the storage period. Values are presented as mean ± SEM (*n* = 3). * *p* < 0.05, ** *p* < 0.01.

Color evolution is a critical visual indicator of oil quality and oxidative status during storage. Therefore, the impact of daidzein fortification on the color stability of soybean oil was evaluated over a six-month storage period at room temperature. Samples were analyzed at the beginning (0 months), at the mid-point (3 months), and at the end of storage (6 months) (Table S2). At time zero, no significant difference (*p* > 0.05) was found between the plain oil (L: 33.06 ± 0.29, a: 0.09 ± 0.01, b: 6.92 ± 0.04) and the fortified oil (L: 32.84 ± 0.05, a: 0.12 ± 0.02, b: 6.80 ± 0.02). After six months of storage, both oils underwent significant color shifts characteristic of oxidation: a decrease in L value (lightness) and an increase in a (redness) and b value (yellowness). However, the extent of deterioration was markedly less in the fortified oil. The daidzein-fortified oil retained a significantly higher L value (30.38 ± 0.09 vs. 26.75 ± 0.09) and a lower a (0.45 ± 0.01 vs. 0.79 ± 0.01) and b value (7.70 ± 0.01 vs. 9.43 ± 0.01) compared to the plain oil (*p* < 0.05). These quantitative data are fully consistent with the visual observations in Fig. 3A. The red and yellowing of soybean oil during storage are direct visual consequences of oxidative degradation, primarily involving the formation of colored secondary oxidation products such as carbonyls and melanoidins and the interaction of tocored compounds and oxidized triacylglycerols (Chen & Sun, 2023). The significantly attenuated color change in the daidzein-fortified oil demonstrates that daidzein effectively retards lipid oxidation. This preservation of color aligns with previous studies on other natural phenolics in oil systems (Li et al., 2022; Li et al., 2023) and supports the dual role of daidzein as both an antioxidant and a color stabilizer in edible oils.

#### 3.2.2 Changes in acid value and peroxide value

To further evaluate oxidative stability beyond color, two fundamental chemical indices of oil quality, acid value (AV) and peroxide value (PV), were monitored throughout the six-month storage period. At the initiation of storage (0 months), the AV and PV of the daidzein-fortified oil were not statistically different from those of the plain soybean oil (*p* > 0.05), confirming a similar initial quality baseline (Fig. 3B and C). However, as storage progressed, marked differences emerged. After six months, the daidzein-fortified oil exhibited a significantly lower final AV (0.17 mg KOH/g) compared to the plain oil (0.21 mg KOH/g) (*p* < 0.05; Fig. 3B). An identical protective trend was observed for PV, where the fortified oil maintained a significantly lower value than the control at the end of the storage period (*p* < 0.05; Fig. 3C).

The increase in AV and PV during storage is directly attributed to the hydrolytic and primary oxidative degradation of triglycerides, leading to the formation of free fatty acids and hydroperoxides, respectively (Chen et al., 2025). The significantly attenuated rise in both indices in the daidzein-fortified oil provides direct chemical evidence that daidzein effectively retards lipid oxidation. This is consistent with its role as a phenolic antioxidant, which likely interrupts the free-radical chain reaction of autoxidation, thereby suppressing the formation of primary oxidation products (hydroperoxides) and their subsequent breakdown into secondary products, including low-molecular-weight acids that contribute to AV (Machado et al., 2023). These results collectively demonstrate that daidzein fortification significantly enhances the oxidative stability of soybean oil during extended storage.

#### 3.2.3 Changes in fatty acid composition

Soybean oil is characterized by a high content (> 80%) of unsaturated fatty acids (UFAs) (Block et al., 2022; Orsavova et al., 2015). During storage, these UFAs are susceptible to oxidative degradation, leading to alterations in the overall fatty acid profile, typically observed as a relative decrease in UFA content and a corresponding increase in saturated fatty acids (SFAs) due to the loss of oxidatively labile unsaturated bonds (Multari et al., 2019). To further elucidate the protective effect of daidzein at the molecular level, its influence on the fatty acid composition of soybean oil during 6-month storage was analyzed at 90-day intervals. As detailed in Table 1, nine fatty acids were identified, with palmitic acid (C16:0), oleic acid (C18:1), and linoleic acid (C18:2) being predominant, consistent with literature (Fu et al., 2026). The total UFA content decreased in both plain and fortified oils over time. However, the extent of this decrease was markedly mitigated by daidzein fortification. By the end of the storage period, the total UFA content in plain soybean oil declined from an initial 79.48 g/100 g to 77.24 g/100 g, representing a loss of 2.24 g/100 g. In contrast, the daidzein-fortified oil showed a significantly smaller reduction from an initial 79.80 g/100 g to 78.48 g/100 g, representing a loss of 1.32 g/100 g. Consequently, the increase in total SFA content was lower in the fortified oil (0.18 g/100 g) compared to the plain oil (0.27 g/100 g). These results clearly demonstrate that daidzein addition effectively slowed the oxidation-driven decline in UFA content, providing direct compositional evidence for its role in enhancing the oxidative stability of soybean oil during storage. The protective effect can be attributed to the established antioxidant mechanisms of phenolic compounds like daidzein. Lipid oxidation is initiated by the formation of fatty acid radicals, which react with oxygen to form peroxyl radicals (ROO•), propagating a chain reaction that yields primary products like hydroperoxides and secondary products such as aldehydes and ketones, ultimately degrading UFAs (Porter, 2025; Yildiz et al., 2025). Daidzein acts primarily by donating hydrogen atoms to these peroxyl radicals, forming more stable isoflavone radicals and thereby terminating the propagation chain (Yildiz et al., 2025). Additionally, its potential metal-chelating capacity may deactivate pro-oxidant transition metal ions, further suppressing oxidation initiation (Mukherjee et al., 2025). By retarding the formation of primary oxidation products (hydroperoxides), daidzein effectively preserves the integrity of unsaturated fatty acids, as corroborated by our compositional data and supported by similar findings for other phenolic antioxidants (Xu et al., 2022).

**Table 1:** Effect of daidzein on the fatty acid profile of soybean oil during storage.

Fatty acids	Storage time (month)					
	0		3		6	
	DZSO	SO	DZSO	SO	DZSO	SO
Palmitic acid (C16:0)	10.71 ± 0.02^c^	10.73 ± 0.01^c^	10.74 ± 0.03^b^	10.79 ± 0.01^b^	10.80 ± 0.02^a^	10.85 ± 0.02^a^
Stearic acid (C18:0)	4.22 ± 0.06^b^	4.26 ± 0.04^c^	4.28 ± 0.03^a^	4.36 ± 0.03^b^	4.30 ± 0.01^a^	4.40 ± 0.02^a^
Oleic acid (C18:1)	23.86 ± 0.09^a^	23.92 ± 0.09^a^	23.67 ± 0.10^b^	23.43 ± 0.06^b^	23.36 ± 0.03^c^	23.00 ± 0.05^c^
Linoleic acid (C18:2)	49.87 ± 0.05^a^	49.55 ± 0.34^a^	49.38 ± 0.07^b^	49.01 ± 0.04^ab^	49.25 ± 0.05^c^	48.66 ± 0.03^b^
Arachidic acid (C20:0)	0.38 ± 0.01^c^	0.39 ± 0.01^c^	0.41 ± 0.02^b^	0.45 ± 0.01^b^	0.40 ± 0.01^a^	0.50 ± 0.01^a^
Linolenic acid (C18:3)	0.35 ± 0.02^a^	0.33 ± 0.01^a^	0.31 ± 0.02^b^	0.28 ± 0.02^b^	0.28 ± 0.01^b^	0.22 ± 0.01^c^
Eicosenoic acid (**C20:1)**	0.84 ± 0.03^a^	0.87 ± 0.02^a^	0.83 ± 0.01^a^	0.82 ± 0.01^b^	0.81 ± 0.02^a^	0.77 ± 0.01^c^
α-Linolenic acid (C18:3 N3)	4.87 ± 0.03^a^	4.82 ± 0.05^a^	4.87 ± 0.02^a^	4.67 ± 0.02^b^	4.78 ± 0.02^b^	4.58 ± 0.02^c^
Behenic acid (C22:0)	0.04 ± 0.00^a^	0.05 ± 0.00^b^	0.05 ± 0.00^a^	0.05 ± 0.01^ab^	0.05 ± 0.01^a^	0.06 ± 0.00^a^
∑SFA	15.36 ± 0.08^c^	15.53 ± 0.04^c^	15.49 ± 0.03^b^	15.65 ± 0.03^b^	15.54 ± 0.03^a^	15.80 ± 0.03^a^
∑MUFA	24.71 ± 0.07^a^	24.78 ± 0.10^a^	24.51 ± 0.09^b^	24.25 ± 0.06^b^	24.17 ± 0.03^c^	23.77 ± 0.05^c^
∑PUFA	55.09 ± 0.06^a^	54.70 ± 0.28^a^	54.56 ± 0.03^b^	53.96 ± 0.04^b^	54.31 ± 0.07^c^	53.47 ± 0.05^c^
∑TFA	95.15 ± 0.11^a^	95.02 ± 0.34^a^	94.55 ± 0.06^b^	93.87 ± 0.13^b^	94.01 ± 0.11^c^	93.04 ± 0.09^c^

Note: Values are presented as mean ± standard deviation *(n = 3)*. SFA, saturated fatty acids; MUFA, monounsaturated fatty acids; PUFA, polyunsaturated fatty acids; TFA, total fatty acids. A *t*-test was used to analyze the statistical differences in fatty acid concentrations between soybean oil with and without daidzein at each time point. Different superscript lowercase letters (a, b) indicate significant differences *(p < 0.05)*.

The efficacy of daidzein aligns with a growing interest in natural antioxidants for food stabilization. While synthetic antioxidants like TBHQ are effective and commonly used, their application is subject to regulatory limits (e.g., 0.02% in oils under FDA regulations) due to safety considerations (Xu et al., 2021). Natural alternatives, including various plant extracts and bioactive compounds, have demonstrated comparable potential in inhibiting lipid oxidation without facing the same stringent restrictions in many jurisdictions (Benbouriche et al., 2024; Pennisi et al., 2023). The results of this study, showing that daidzein effectively preserves fatty acid composition and other quality indices, position soybean isoflavones as promising natural candidates for developing healthier and more stable lipid products.

#### 3.2.4 Stability of daidzein during oil storage

**Fig. 4:**
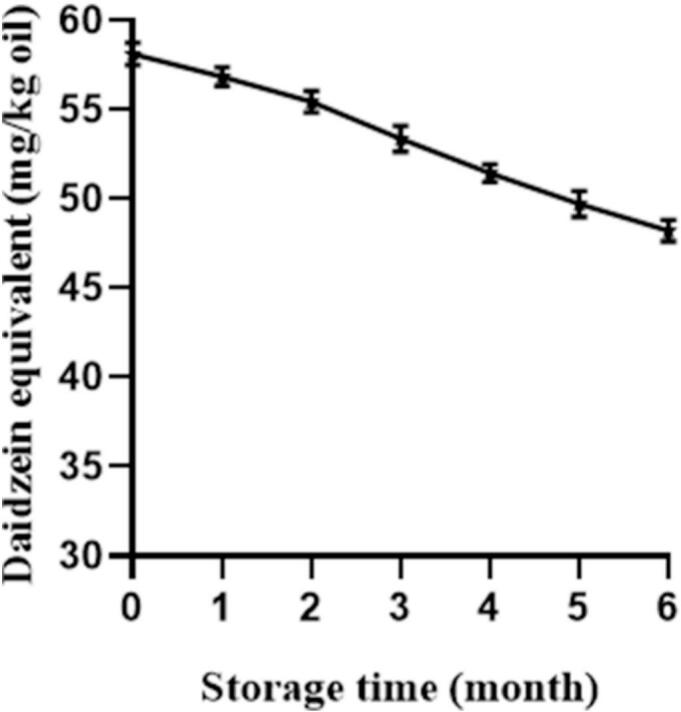
Stability of daidzein in fortified soybean oil during storage. Changes in the concentration of daidzein monitored monthly over a six-month storage period at room temperature. Values are presented as mean ± SEM (n = 3).

The inherent stability of the fortified additive is crucial for its functionality. Therefore, the concentration of daidzein in the fortified soybean oil was monitored monthly over the 6-month storage period at room temperature (Fig. 4). A gradual decrease in daidzein content was observed throughout storage. The initial concentration of 58.12 mg/kg declined to 48.22 mg/kg after six months, corresponding to a net loss of approximately 17.03%. This steady reduction suggests the consumption of daidzein, likely as it performs its antioxidant role by scavenging free radicals generated during the slow, autoxidative degradation of the oil matrix.

The degradation profile of daidzein can be contextualized by comparing it with synthetic antioxidants. For instance, it is reported that in soybean oil stored under similar conditions, the synthetic antioxidant tert-butylhydroquinone (TBHQ) at an initial concentration of 200 mg/kg degraded by 9.5% within two months, with its primary oxidation product, tert-butylquinone (TQ), accounting for most of the loss (Li et al., 2014; Liu et al., 2016). In the present study, daidzein exhibited a degradation rate of approximately 5.37% over a two-month period. While a direct quantitative comparison is constrained by differing initial concentrations and oil type, this trend indicates that daidzein possesses considerable stability in oil during ambient storage, comparable to the behavior reported for some synthetic antioxidants under similar conditions. It is important to note that a direct, head-to-head comparison between daidzein and established antioxidants (e.g., α-tocopherol, rosemary extract, TBHQ) under identical conditions was not performed in this study. Therefore, the data should not be interpreted as evidence of daidzein's superiority over these alternatives. Future research should include such comparative evaluations to better contextualize the practical advantages of daidzein fortification. This stability during storage is a positive attribute for a functional additive. However, as thermal processing represents a more intense oxidative challenge common in oil use, evaluating the stability of daidzein under such conditions is essential for a comprehensive assessment of its practical utility, which is addressed in the following section.

### 3.3 Thermal loss of daidzein in a French fry frying model

#### 3.3.1 Effect of heating time and temperature on daidzein retention

**Fig. 5:**
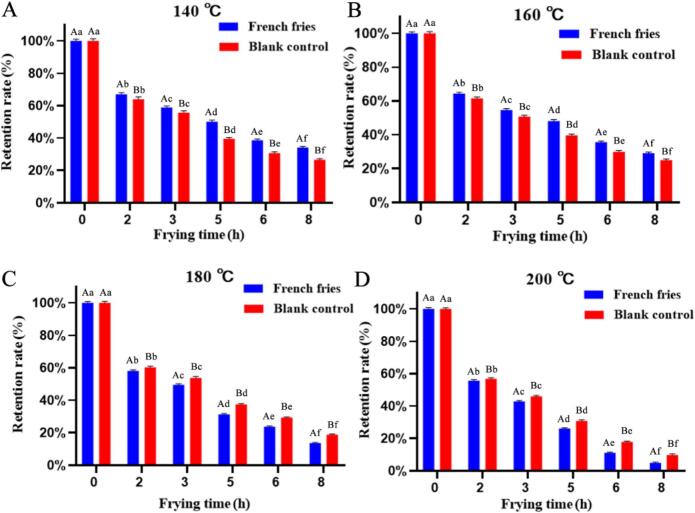
Effect of frying time and temperature on the retention rate of daidzein in oil during a simulated French fry process. Panels (A–D) show daidzein retention in the oil phase at frying temperatures of 140, 160, 180, and 200 °C, respectively. Data are presented as mean ± SEM (n = 3). Uppercase letters (A, B) denote significant differences between the treatments (with vs. without French fries) at the same time point, while lowercase letters (a-f) denote significant differences across different frying times for the same treatment.

Edible oils are frequently subjected to thermal processing such as frying. To assess the thermal stability of daidzein under such practical conditions, its degradation was evaluated in a controlled experimental batch-frying model. The results are expressed as the daidzein retention rate in the oil phase. As shown in Fig. 5, the retention of daidzein progressively decreased with prolonged heating time at all four frying temperatures tested (140, 160, 180, and 200 °C). Notably, across all temperatures, more than half of the total daidzein loss occurred after approximately five hours of cumulative frying time. This accelerated degradation phase likely coincides with the depletion of endogenous antioxidants, such as native tocopherols, inherently present in the soybean oil. During the initial stages of frying, these native antioxidants may preferentially scavenge free radicals, temporarily shielding the added daidzein. However, once this native protective pool is exhausted, marking the end of the oil's induction period, daidzein is subjected to the full extent of the thermal and oxidative stress, leading to a rapid and pronounced decline in its retention (Xu et al., 2025). Furthermore, a distinct and temperature-dependent pattern emerged when comparing daidzein retention in oil used for frying French fries versus oil heated alone (control). At the lower frying temperatures of 140 °C and 160 °C, the presence of French fries led to a significantly higher daidzein retention in the oil compared to the control (Fig. 5A, B). Conversely, at the higher temperatures of 180 °C and 200 °C, this relationship reversed: daidzein retention in the oil from the French fry system became significantly lower than in the control oil heated without food (Fig. 5C, D). This reversal suggests a shift in the dominant factors influencing daidzein stability with increasing temperature. At lower temperatures (140–160 °C), the high moisture content of the French fries likely exerts a protective effect. The rapid vaporization of water at the food-oil interface creates a local steam blanket, which can moderate the local temperature and reduce oxygen availability, thereby attenuating the autocatalytic lipid oxidation that consumes antioxidants like daidzein (Chiou et al., 2013; Li et al., 2023). At elevated temperatures (180–200 °C), this protective mechanism diminishes as moisture is rapidly expelled. Simultaneously, several intensifying degradative pathways become predominant. The severe thermal stress significantly accelerates Maillard reactions and thermal decomposition of food components, generating a high concentration of free radicals and reactive carbonyl species that can directly attack and degrade daidzein. Furthermore, increased leaching of pro-oxidant metal ions from the food matrix into the oil may further catalyze oxidation reactions (Multari et al., 2019). In this high-intensity oxidative environment, daidzein is rapidly consumed through its radical-scavenging activity, leading to a net retention lower than in the simpler, food-free control system where such reactive species are less abundant (Mukherjee et al., 2025). This complex interaction between food components and frying temperature critically defines the fate of daidzein during thermal processing.

#### 3.3.2 Thermal loss pathways of daidzein in soybean oil

To elucidate the mechanisms underlying the reduced retention of daidzein during frying, we systematically investigated its potential loss pathways, guided by reports that antioxidants in oils can be depleted through volatilization, chemical transformation, and migration into food during thermal processing (Li et al., 2023; Santos et al., 2012).

**1) Volatilization.** To assess potential volatilization loss, daidzein-fortified oil was heated at 180 °C for 4 h under reflux condensation. White crystals deposited at the cooler neck of the flask were collected and analyzed by HPLC. As shown in Fig. S2, these crystals exhibited an identical retention time and UV spectrum to the daidzein standard, confirming that volatilization and subsequent re-condensation contribute to daidzein loss during heating.

**Fig. 6:**
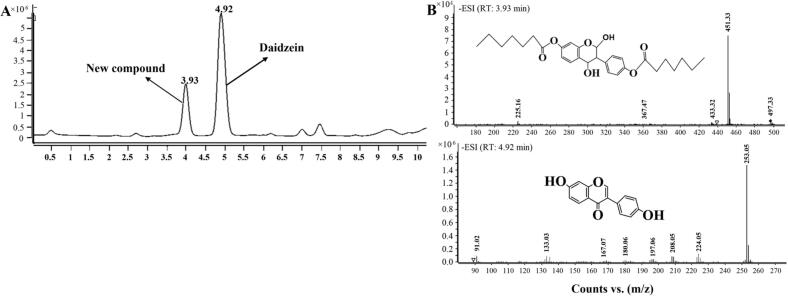
UPLC-Q-TOF-MS analysis (ESI^−^ mode) of daidzein-fortified soybean oil after heating at 180 °C for 8 h. (A) Total ion chromatogram (TIC). (B) MS spectra corresponding to the major peaks in the TIC: (i) the peak at Rt 3.93 min (Compound I, [M-H]^−^ at *m/z* 497) and (ii) the peak at Rt 4.92 min (Compound II, [M-H]^−^ at *m/z* 253).

**2) Chemical Transformation.** To identify non-volatile transformation products, oil heated at 180 °C for 8 h was analyzed by UPLC-Q-TOF-MS/MS. The chromatogram (Fig. 6A) revealed two major peaks at retention times (Rt) of 3.93 min and 4.92 min. The peak at Rt 4.92 min yielded a deprotonated molecular ion [M-H]^−^ at *m/z* 253, consistent with intact daidzein (MW 254), which was confirmed by its characteristic fragment ions at *m/z* 91, 133, 208, and 224 (Fig. 6B) matching literature data (Fallas-Ramirez et al., 2018; Sun et al., 2025). The earlier eluting peak at Rt 3.93 min corresponded to a compound with [M-H]^−^ at *m/z* 497 (MW 498). Collision-induced dissociation (CID) of this precursor produced characteristic fragment ions at *m/z* 451, 433, 367, and 225. Notably, the *m/z* 225 fragment is consistent with the cleavage of the daidzein core, strongly suggesting that the original daidzein skeleton is retained within this larger molecule. Regarding the substantial mass increase, we hypothesize that this unidentified transformation product formed via the reaction of daidzein with lipid oxidation intermediates generated during the extended heating of the oil. This hypothesis is analogous to thermal behaviors observed in other flavonoids; for instance, Zhang et al. (2021) reported that quercetin can capture lipid oxidation intermediates to form ester derivatives. While the MS/MS data and literature precedent align with the formation of a daidzein-lipid adduct, precise structural elucidation requires further targeted investigation, such as isolation and NMR spectroscopy. Consequently, this compound is cautiously classified as an unidentified daidzein-derived thermal transformation product.

**Fig. 7:**
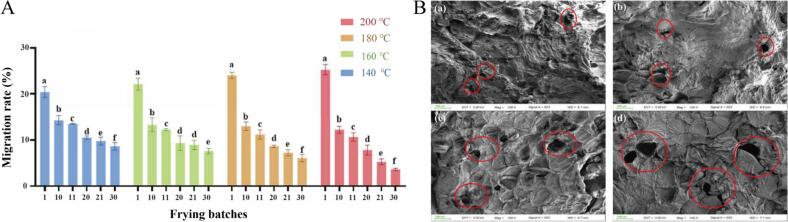
Effect of frying temperature and batch sequence on the migration of daidzein from oil into French fries and the associated structural changes in the fry crust. (A) Migration rate of daidzein into French fries over successive frying batches at four temperatures: 140, 160, 180, and 200 °C. Data are presented as mean ± SEM (n = 3). The values not sharing a common letter (a-f) are significantly different (*p* < 0.05). (B) Corresponding scanning electron micrographs (SEM) of the crust microstructure of French fries fried at (a) 140, (b) 160, (c)180, and (d) 200 °C (magnification: 100×).

**3) Migration into Food.** The migration of daidzein from the frying oil into French fries was quantified across multiple frying cycles (batches) at four temperatures (Fig. 7A). To properly interpret this migration behavior, it is essential to consider the coupled dynamics of moisture loss and total oil uptake. During deep-frying, intense heat causes the rapid evaporation of moisture from the potato. As water escapes, it creates a porous crust structure. Subsequently, driven by capillary action and the pressure differential created as internal steam condenses, the food matrix absorbs the surrounding oil. Since daidzein is dissolved in the oil phase, this bulk oil uptake serves as the primary physical transport mechanism carrying daidzein into the fry matrix. In the initial frying batch, higher temperatures promoted greater migration (e.g., 25.26% at 200 °C vs. 20.40% at 140 °C). This initial increase is directly attributed to the more aggressive moisture loss at elevated temperatures, which in turn drives a higher volume of initial oil uptake and accelerates mass transfer kinetics. However, with successive batches, the migration rate declined more rapidly at higher temperatures. By the 30th batch, the migration rate at 200 °C had dropped to 3.65%, while at 140 °C it remained at 8.67%. This attenuation is linked to the concurrent depletion of daidzein from the oil via volatilization and degradation, as previously described, and to the progressive structural changes in the food matrix. Scanning electron microscopy (SEM) provided supporting visual observations that the crust of fries fried at higher temperatures developed larger pores and fissures (Fig. 7B). While these enlarged pores, formed by accelerated water evaporation and starch gelatinization (Shen et al., 2022), initially act as efficient capillary channels for oil and dissolved daidzein uptake, the continuous depletion of the bulk daidzein concentration in the oil means less bioactive compound is available to be transported per unit of oil absorbed in later batches. This phenomenon aligns with reports on the transfer of other heat-sensitive compounds, such as olive oil polyphenols, into fried foods (Chiou et al., 2013).

Collectively, the thermal loss of daidzein during the French fry frying process occurs via three interconnected pathways: volatilization, chemical transformation into an unidentified daidzein-derived product, and migration into the food matrix. The dominance and interplay of these pathways are profoundly influenced by frying temperature and duration, as well as the dynamic physical changes in the food. These findings suggest that a “gentle frying” strategy, employing moderately lower temperatures and shorter durations, could help better preserve functional components like daidzein both in the oil and in the final fried product, offering practical guidance for optimizing nutritional quality in thermal food processing.

Despite the significant findings presented, this study has certain limitations that warrant further investigation. Firstly, the present study focused primarily on the fate and thermal loss kinetics of daidzein during the frying model, and thus the PV and AV of the fried oil samples at different temperatures were not measured; tracking these indices would provide a more complete picture of the oil's oxidative state during frying. Secondly, while volatilization, chemical transformation, and food migration were identified as contributing routes to daidzein loss, a strictly closed, quantitative mass balance was not achieved. Future research should aim to precisely quantify the daidzein distribution across all phases, including a comprehensive recovery of volatile condensates. Thirdly, the definitive structural elucidation of the tentatively identified daidzein-derived transformation product (*m/z* 497) requires advanced analytical techniques, such as NMR spectroscopy on the isolated compound. Fourth, while we propose that the undissolved daidzein residue can be recovered and recycled, the actual recovery yield, purity, and reusability across multiple batches have not been experimentally verified. The impact of ultrasonication on particle morphology and crystallinity, which may affect re-dissolution efficiency, also remains to be characterized. These aspects are important directions for future process optimization. Looking forward, the green, solvent-free physical solubilization strategy developed herein possesses broad application potential. We hypothesize that this ultrasound-assisted magnetic stirring method can be successfully extrapolated to enrich various other edible oils with different moderately lipophobic bioactive flavonoids, such as quercetin or genistein. However, applying this method to new systems will necessitate the re-optimization of key processing parameters, particularly the initial solid loading and ultrasonication conditions, to accurately accommodate specific solute-solvent interactions.

## 4 Conclusion

In this study, the solubility of daidzein in soybean oil was effectively enhanced by a solvent-free, ultrasound-assisted magnetic stirring method, resulting in a fortified oil with a daidzein concentration of 58.12 mg/kg. The incorporation of daidzein significantly improved the oxidative stability of the oil during six months of storage, as evidenced by the attenuated deterioration in color, lower acid and peroxide values, and better preservation of unsaturated fatty acids compared to unfortified oil. Furthermore, employing a French fry frying model revealed the complex thermal behavior of daidzein. Its loss during frying was governed by three contributing pathways, volatilization, chemical transformation into an unidentified daidzein-derived product, and migration into the food matrix, with the extent and dominance of each pathway being critically dependent on frying temperature, duration, and associated changes in food microstructure. Given its documented bioactivity, the demonstrated efficacy in stabilizing soybean oil, and the characterized kinetics of its degradation and transfer during thermal processing, daidzein presents considerable potential as a natural multifunctional additive for edible oils. The findings of this work provide a foundational understanding and practical insights for the development of daidzein-fortified oil products and offer guidance for optimizing thermal processing parameters to better retain bioactive components in fried foods.

## CRediT authorship contribution statement

**Tan Wang:** Writing – original draft, Methodology, Investigation. **Siwei Chen:** Writing – original draft, Methodology, Investigation. **Wenxi Li:** Methodology, Investigation. **Maofeng Dong:** Methodology. **Shiquan Shen:** Writing – review & editing. **Mingfu Wang:** Writing – review & editing. **Hui Wang:** Writing – review & editing. **Yueliang Zhao:** Writing – review & editing, Supervision, Funding acquisition, Conceptualization.

## Declaration of competing interest

The authors declare that they have no known competing financial interests or personal relationships that could have appeared to influence the work reported in this paper.

## Data Availability

Data will be made available on request.

## References

[bb0005] Ahmad S., Ahsan F., Ansari J.A., Mahmood T., Bano S., Shahanawaz M. (2024). Bioflavonoid daidzein: Therapeutic insights, formulation advances, and future directions. Drug Research.

[bb0010] Benbouriche A., Haddadi-Guemghar H., Mehidi-Terki D., Boulekbache-Makhlouf L., Bachir-bey M. (2024). Enrichment of soybean oil with carotenoids from chili paste by-product: Enhancement of quality, oxidative stability, and thermo-resistance. Journal of Food Measurement and Characterization.

[bb0015] Block J.M., Danielski R., Teixeira G.L., Nunes I.L. (2022). Phytochemicals in Soybeans.

[bb0020] Chaaban H., Ioannou I., Chebil L., Slimane M., Gérardin C., Paris C., Ghoul M. (2017). Effect of heat processing on thermal stability and antioxidant activity of six flavonoids. Journal of Food Processing and Preservation.

[bb0025] Chen S., Ge C., Mu B., Zhao C., Wang J., Li H., Li G. (2025). Effects of onion skin extract on lipid oxidation and volatile flavor compounds of low-salt dry-cured ham during ripening. LWT.

[bb0030] Chen X., Sun S. (2023). Color reversion of refined vegetable oils: A review. Molecules.

[bb0035] Cheng M., He J., Li C., Wu G., Zhu K., Chen X., Tan L. (2023). Comparison of microwave, ultrasound and ultrasound-microwave assisted solvent extraction methods on phenolic profile and antioxidant activity of extracts from jackfruit (*Artocarpus heterophyllus* lam.) pulp. LWT.

[bb0040] Chiou A., Kalogeropoulos N., Efstathiou P., Papoutsi M., Andrikopoulos N.K. (2013). French fries oleuropein content during the successive deep frying in oils enriched with an olive leaf extract. International Journal of Food Science and Technology.

[bb0045] Erdoğan Orhan İ.L.K.A.Y., Oezcelik B., Kartal M., Aslan S., Sener B., Özgüven M. (2007). Quantification of daidzein, genistein and fatty acids in soybeans and soy sprouts, and some bioactivity studies. Acta Biologica Cracoviensia Series Botanica.

[bb0050] Fallas-Ramirez J.M., Hernandez L., Vaillant F. (2018). Untargeted metabolomic profiling of urine in Wistar rats reveals enhanced bioavailability of soy isoflavones post short-term consumption of noni (Morinda citrifolia) juice. Journal of Functional Foods.

[bb0055] Fu G., Yan Y., Li Y., Zhang Z., Wang M., Zhao Y. (2026). Genistein-7-O-octanoate: A promising natural antioxidant for stabilizing soybean oil during storage and high-temperature frying. Food Research International.

[bb0060] Kong L., Fan X., Guo L., Jiang Q., Xiao J., Fan D., Zhao Y. (2023). Effects of stigmasterol on 3-chloropropane-1, 2-diol fatty acid esters and aldehydes formation in heated soybean oil. Journal of Agricultural and Food Chemistry.

[bb0065] Lee S.J., Ahn J.K., Kim S.H., Kim J.T., Han S.J., Jung M.Y., Chung I.M. (2003). Variation in isoflavone of soybean cultivars with location and storage duration. Journal of Agricultural and Food Chemistry.

[bb0070] Li J., Bi Y., Liu W., Sun S., Liu C., Ma S. (2014). Effect of acid value on TBHQ and BHT losses in heating oils: Identification of the esterification products of TBHQ and free fatty acids. Journal of the American Oil Chemists’ Society.

[bb0075] Li J., Zhang S., Kuang Y., Bi Y., Wang H. (2023). A review on losses and transformation mechanisms of common antioxidants. Journal of the American Oil Chemists' Society.

[bb0080] Li T., Guo Q., Qu Y., Li Y., Liu H., Liu L., Wang Q. (2022). Solubility and physicochemical properties of resveratrol in peanut oil. Food Chemistry.

[bb0085] Liu C., Li J., Bi Y., Wang X., Sun S., Yang G. (2016). Thermal losses of tertiary butylhydroquinone (TBHQ) and its effect on the qualities of palm oil. Journal of Oleo Science.

[bb0090] Liu S., Zhu Y., Liu N., Fan D., Wang M., Zhao Y. (2021). Antioxidative properties and chemical changes of quercetin in fish oil: Quercetin reacts with free fatty acids to form its ester derivatives. Journal of Agricultural and Food Chemistry.

[bb0095] Machado M., Rodriguez-Alcalá L.M., Gomes A.M., Pintado M. (2023). Vegetable oils oxidation: Mechanisms, consequences and protective strategies. Food Reviews International.

[bb0100] Mukherjee B., Al Hoque A., Hota S.H., Gope S., Ray M., Barman M., Das L. (2025). Dietary supplements and nutraceuticals.

[bb0105] Multari S., Marsol-Vall A., Heponiemi P., Suomela J.P., Yang B. (2019). Changes in the volatile profile, fatty acid composition and other markers of lipid oxidation of six different vegetable oils during short-term deep-frying. Food Research International.

[bb0110] Orsavova J., Misurcova L., Vavra Ambrozova J., Vicha R., Mlcek J. (2015). Fatty acids composition of vegetable oils and its contribution to dietary energy intake and dependence of cardiovascular mortality on dietary intake of fatty acids. International Journal of Molecular Sciences.

[bb0115] Pennisi R., Ben Amor I., Gargouri B., Attia H., Zaabi R., Chira A.B., Sciortino M.T. (2023). Analysis of antioxidant and antiviral effects of olive (*Olea europaea* L.) leaf extracts and pure compound using cancer cell model. Biomolecules.

[bb0120] Porter N.A. (2025). Chemical mechanisms of lipid peroxidation. Redox Biochemistry and Chemistry.

[bb0125] Santos N.A., Cordeiro A.M., Damasceno S.S., Aguiar R.T., Rosenhaim R., Carvalho J.R., Souza A.G. (2012). Commercial antioxidants and thermal stability evaluations. Fuel.

[bb0130] Shah Y., Zhou X., Tang J., Takhar P.S. (2025). The effect of conventional and microwave frying on the quality characteristics of French fries. Journal of Food Science.

[bb0135] Shen M., Liu X., Xu X., Wu Y., Zhang J., Liang L., Liu G. (2022). Migration and distribution of pah4 in oil to french fries traced using a stable isotope during frying. Journal of Agricultural and Food Chemistry.

[bb0140] Sun Z., Dong F., Zhang R., Song X., Huang X., Dong Y., Wang M. (2025). UPLC-HDMS revealed numerous novel compounds in soybean crude oil. Food Chemistry.

[bb0145] Swallah M.S., Yang X., Li J., Korese J.K., Wang S., Fan H., Huang Q. (2023). The pros and cons of soybean bioactive compounds: An overview. Food Reviews International.

[bb0150] Vojvodić S., Čonić B.S., Torović L. (2023). Safety assessment of herbal food supplements: Ethanol and residual solvents associated risk. Journal of Food Composition and Analysis.

[bb0155] Wang S., Li Y., Ma C., Huang D., Chen S., Zhu S., Wang H. (2023). Enzymatic molecular modification of water-soluble polyphenols: Synthesis, structure, bioactivity and application. Critical Reviews in Food Science and Nutrition.

[bb0160] Wang T., Dong M., Shen Q., Wen G., Wang M., Zhao Y. (2024). Development of a UPLC-MS/MS-based method for simultaneous determination of advanced glycation end products and heterocyclic amines in stewed meat products. Food Chemistry.

[bb0165] Wu G., Chang C., Hong C., Zhang H., Huang J., Jin Q., Wang X. (2019). Phenolic compounds as stabilizers of oils and antioxidative mechanisms under frying conditions: A comprehensive review. Trends in Food Science & Technology.

[bb0170] Xu L., Mei X., Wu G., Karrar E., Jin Q., Wang X. (2022). Inhibitory effect of antioxidants on key off-odors in French fries and oils and prolong the optimum frying stage. LWT.

[bb0175] Xu L., Yu J., Wei P., Pan M., Pang B., Liu Z., Li D. (2025). Exploring the effect of frying process on the optimum flavor range of French fries by monitoring changes in total polar compounds. Food Research International.

[bb0180] Xu X., Liu A., Hu S., Ares I., Martínez-Larrañaga M.R., Wang X., Martínez M.A. (2021). Synthetic phenolic antioxidants: Metabolism, hazards and mechanism of action. Food Chemistry.

[bb0185] Yildiz A.Y., Öztekin S., Anaya K. (2025). Effects of plant-derived antioxidants to the oxidative stability of edible oils under thermal and storage conditions: Benefits, challenges and sustainable solutions. Food Chemistry.

[bb0190] Zhang X., Ni L., Zhu Y., Liu N., Fan D., Wang M., Zhao Y. (2021). Quercetin inhibited the formation of lipid oxidation products in thermally treated soybean oil by trapping intermediates. Journal of Agricultural and Food Chemistry.

[bb0195] Zhao X., Ma F., Li P., Li G., Zhang L., Zhang Q., Wang X. (2015). Simultaneous determination of isoflavones and resveratrols for adulteration detection of soybean and peanut oils by mixed-mode SPE LC–MS/MS. Food Chemistry.

[bb0200] Zhao Y., Zhang X., Zhang N., Zhou Q., Fan D., Wang M. (2022). Lipophilized apigenin derivatives produced during the frying process as novel antioxidants. Food Chemistry.

