# Supplementary material for: Enhancing the solubility of daidzein in soybean oil via ultrasound-assisted magnetic stirring: Impacts on oil storage stability and thermal loss during French fries preparation
Source: Food Chem X. 2026 May 15;36:103987. doi: 10.1016/j.fochx.2026.103987 (PMC13202252; doi:10.1016/j.fochx.2026.103987)
Supplement: Supplementary file 1 — Supplementary material [file mmc1.docx]

**
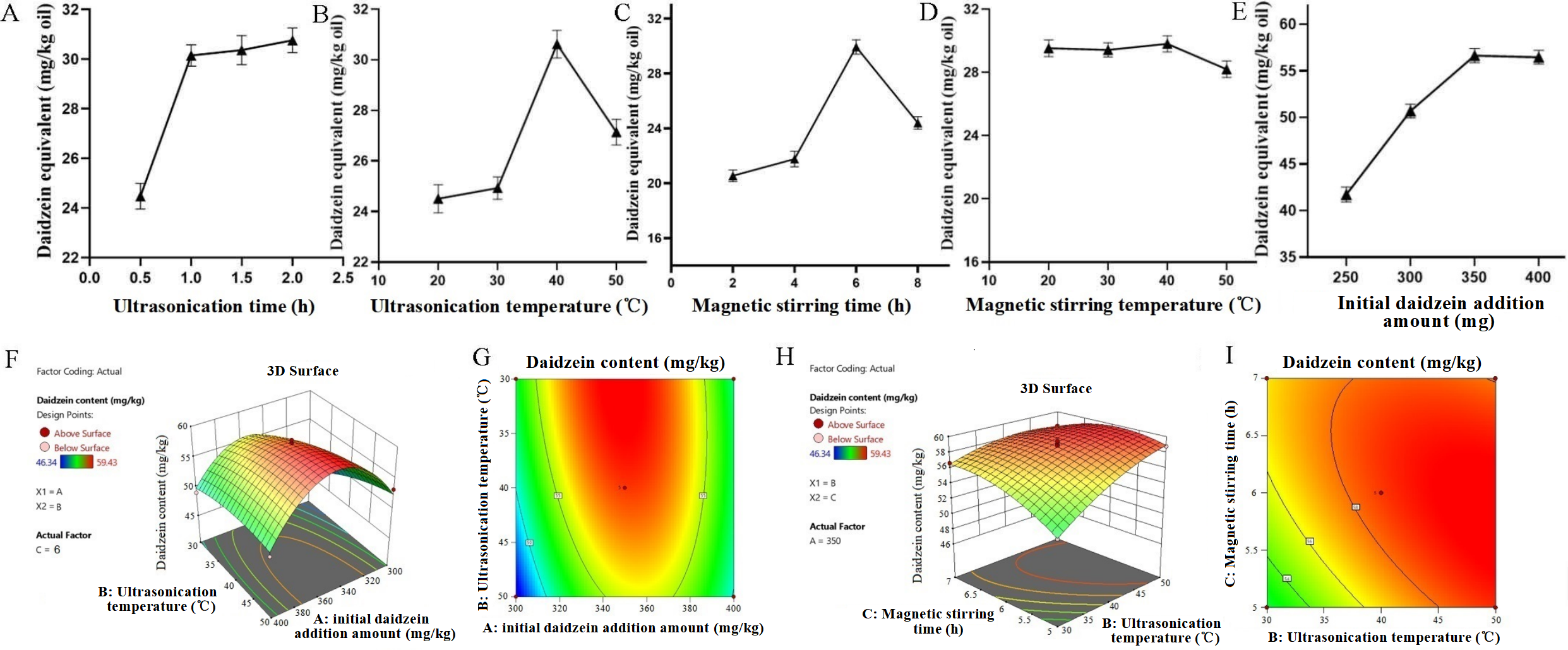
**

Fig. S1 Effect of key processing parameters on daidzein solubility via ultrasound-assisted magnetic stirring. Single-factor effects of (A) ultrasonication time, (B) ultrasonication temperature, (C) magnetic stirring time, (D) magnetic stirring temperature, and (E) initial daidzein addition amount. Response surface (F) and contour (G) plots illustrating the interaction between initial daidzein addition amount (Factor A) and ultrasonication temperature (Factor B). Corresponding response surface (H) and contour (I) plots for the interaction between ultrasonication temperature (Factor B) and magnetic stirring time (Factor C).


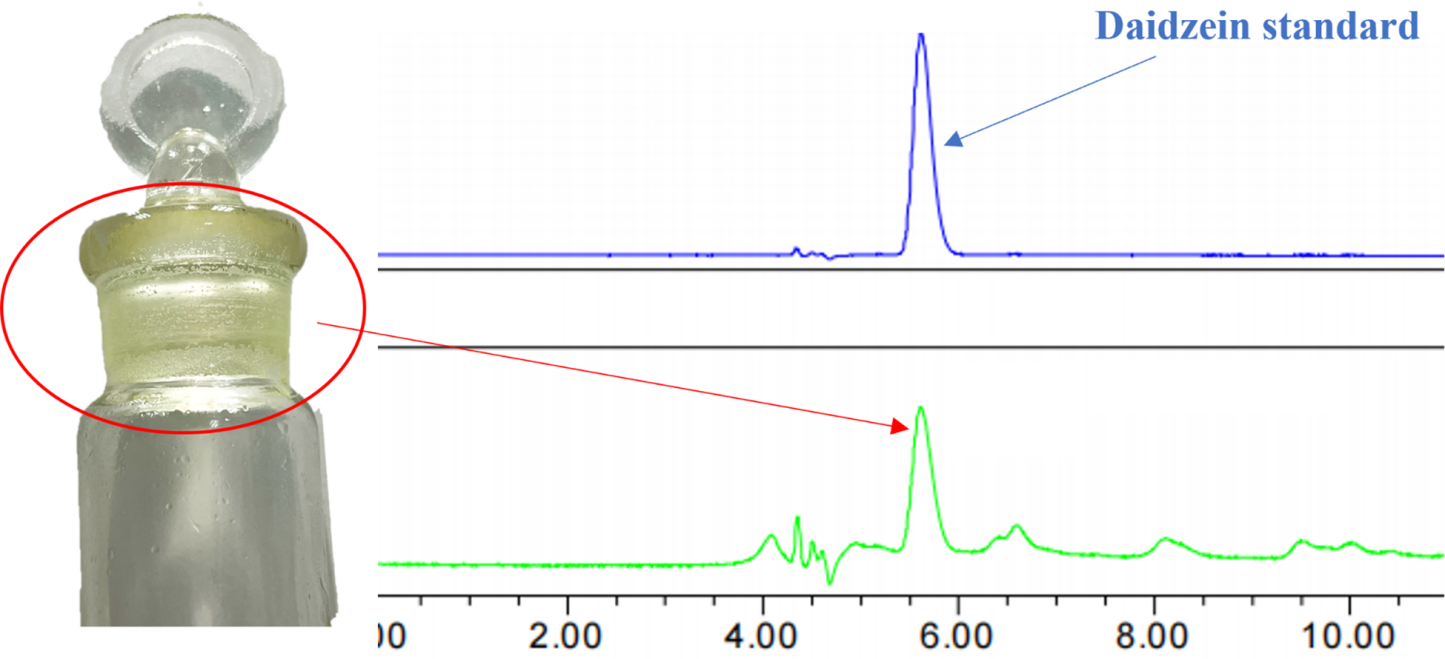


Fig. S2 HPLC analysis of condensable volatiles from heated daidzein-fortified oil.

Table S1a Design matrix and experimental results of the three-factor, three-level Box-Behnken design used to optimize daidzein dissolution in soybean oil via ultrasound-assisted magnetic stirring. Independent variables include: daidzein addition amount (X_1_), ultrasonication temperature (X_2_), and magnetic stirring time (X_3_). Values for the response variable (Y) represent the dissolved daidzein concentration (mg/kg) obtained under each experimental run.

| Number | X1 | X2 | X3 | Y |
| --- | --- | --- | --- | --- |
| 1 | 1 | 0 | 1 | 51.62 |
| 2 | -1 | 0 | 1 | 48.79 |
| 3 | 0 | -1 | 1 | 56.63 |
| 4 | 0 | 0 | 0 | 58.86 |
| 5 | 0 | 0 | 0 | 57.96 |
| 6 | 1 | 0 | -1 | 50.22 |
| 7 | 0 | 1 | -1 | 58.77 |
| 8 | 0 | 0 | 0 | 59.43 |
| 9 | 0 | 0 | 0 | 59.03 |
| 10 | -1 | 0 | -1 | 47.24 |
| 11 | 1 | 1 | 0 | 50.42 |
| 12 | 1 | -1 | 0 | 49.04 |
| 13 | 0 | 1 | 1 | 57.99 |
| 14 | 0 | 0 | 0 | 58.26 |
| 15 | -1 | 1 | 0 | 53.25 |
| 16 | 0 | -1 | -1 | 51.99 |
| 17 | -1 | -1 | 0 | 46.34 |

Table S1b Analysis of variance (ANOVA) for the fitted quadratic response surface model describing the relationship between processing parameters and dissolved daidzein concentration. The table presents the sum of squares, degrees of freedom (df), mean square, F-value, and corresponding *p*-value for the model terms, as well as the lack-of-fit test. * *p* < 0.05, ** *p* < 0.01.

| Source | Sum of Squares | df | Mean square | F-value | *p*-value |
| --- | --- | --- | --- | --- | --- |
| Model | 345.11 | 8 | 43.14 | 58.19 | < 0.0001 |
| X_1_ | 4.03 | 1 | 4.03 | 5.44 | 0.048 |
| X_2_ | 33.74 | 1 | 33.74 | 45.52 | 0.0001 |
| X_3_ | 5.8 | 1 | 5.8 | 7.82 | 0.0233 |
| X_1_X_2_ | 7.65 | 1 | 7.65 | 10.31 | 0.0124 |
| X_2_X_3_ | 7.34 | 1 | 7.34 | 9.91 | 0.0137 |
| X_1_² | 263.54 | 1 | 263.54 | 355.5 | < 0.0001 |
| X_2_² | 4.5 | 1 | 4.5 | 6.07 | 0.0391 |
| X_3_² | 7.44 | 1 | 7.44 | 10.03 | 0.0132 |
| Residual | 5.93 | 8 | 0.7413 |  |  |
| Lack of Fit | 4.52 | 4 | 1.13 | 3.21 | 0.1424 |
| Pure Error | 1.41 | 4 | 0.3521 |  |  |
| Cor Total | 351.04 | 16 |  |  |  |

Table S2 Color parameters (L, a, b) of plain and daidzein-fortified (DZSO) soybean oil during a six-month storage period at room temperature. Color values were measured monthly and are presented as mean ± standard deviation (n = 3). Different superscript lowercase letters within the same column (for each oil type) indicate significant differences (*p* < 0.05) across storage time.

| Storage time (month) | L | | a* | | b* | |
| --- | --- | --- | --- | --- | --- | --- |
|  | DZSO | SO | DZSO | SO | DZSO | SO |
| 0 | 32.84±0.05a | 33.06±0.29a | 0.12±0.02e | 0.09±0.01e | 6.80±0.02f | 6.92±0.04e |
| 1 | 32.76±0.26a | 31.39±0.23b | 0.18±0.02e | 0.17±0.01d | 7.01±0.02e | 7.60±0.04d |
| 2 | 31.73±0.16b | 30.13±0.32c | 0.24±0.02d | 0.25±0.02cd | 7.26±0.04d | 8.50±0.08c |
| 3 | 31.63±0.61b | 29.17±0.27cd | 0.31±0.01c | 0.35±0.01c | 7.28±0.03c | 8.64±0.16c |
| 4 | 31.18±0.09bc | 28.29±0.64de | 0.36±0.01bc | 0.49±0.03b | 7.41±0.01c | 9.08±0.09b |
| 5 | 30.93±0.05c | 27.25±0.08ef | 0.41±0.01ab | 0.64±0.02ab | 7.60±0.01b | 9.27±0.02ab |
| 6 | 30.38±0.09d | 26.75±0.09f | 0.45±0.01a | 0.79±0.01a | 7.70±0.01a | 9.43±0.01a |
